# Characterisation of Early Positive *mcr-1* Resistance Gene and Plasmidome in *Escherichia coli* Pathogenic Strains Associated with Variable Phylogroups under Colistin Selection

**DOI:** 10.3390/antibiotics10091041

**Published:** 2021-08-25

**Authors:** Guerrino Macori, Scott V. Nguyen, Ankita Naithani, Daniel Hurley, Li Bai, Farid El Garch, Frédérique Woehrlé, Christine Miossec, Benjamin Roques, Peadar O’Gaora, James L. Bono, Séamus Fanning

**Affiliations:** 1 UCD-Centre for Food Safety, UCD School of Public Health, Physiotherapy and Sports Science, University College Dublin, Belfield, D04 N2E5 Dublin, Ireland; scott.nguyen@dc.gov (S.V.N.); ankitanaithani261@gmail.com (A.N.); daniel.hurley@ucd.ie (D.H.); li.bai@ucd.ie (L.B.); 2 Public Health Laboratory, District of Columbia Department of Forensic Sciences, Washington, DC 20024, USA; 3 United States Meat Animal Research Center, United States Department of Agriculture (USDA), State Spur 18D, Clay Center, NE 68938, USA; jim.bono@usda.gov; 4 China National Centre for Food Safety & Risk Assessment (CFSA), Microbiology Laboratory, No. 7 Panjiayuan Nanli, Chaoyang District, Beijing 100021, China; 5 Vétoquinol SA, BP 189 CEDEX, 70204 Lure, France; farid.elgarch@vetoquinol.com (F.E.G.); frederique.woehrle@vetoquinol.com (F.W.); c.miossec@vetoquinol.com (C.M.); 6 Conway BioMolecular Institute, UCD School of Biomolecular & Biomedical Science, University College Dublin, Belfield, D04 N2E5 Dublin, Ireland; benjamin.roques@ucdconnect.ie (B.R.); peadar.ogaora@ucd.ie (P.O.); 7 Institute for Global Food Security, Queen’s University Belfast, 19 Chlorine Gardens, Belfast BT9 5DL, UK

**Keywords:** antimicrobial resistance, *Escherichia coli*, colistin resistance, whole genome sequencing, *mcr-1* gene, plasmid

## Abstract

An antibiotic susceptibility monitoring programme was conducted from 2004 to 2010, resulting in a collection of 143 *Escherichia coli* cultured from bovine faecal samples (diarrhoea) and milk-aliquots (mastitis). The isolates were subjected to whole-genome sequencing and were distributed in phylogroups A, B1, B2, C, D, E, and G with no correlation for particular genotypes with pathotypes. In fact, the population structure showed that the strains belonging to the different phylogroups matched broadly to ST complexes; however, the isolates are randomly associated with the diseases, highlighting the necessity to investigate the virulence factors more accurately in order to identify the mechanisms by which they cause disease. The antimicrobial resistance was assessed phenotypically, confirming the genomic prediction on three isolates that were resistant to colistin, although one isolate was positive for the presence of the gene *mcr-1* but susceptible to colistin. To further characterise the genomic context, the four strains were sequenced by using a single-molecule long read approach. Genetic analyses indicated that these four isolates harboured complex and diverse plasmids encoding not only antibiotic resistant genes (including *mcr-1* and *bla*) but also virulence genes (siderophore, ColV, T4SS). A detailed description of the plasmids of these four *E. coli* strains, which are linked to bovine mastitis and diarrhoea, is presented for the first time along with the characterisation of the predicted antibiotic resistance genes. The study highlighted the diversity of incompatibility types encoding complex antibiotic resistance elements such as Tn*6330*, IS*Ecp1*, Tn*6029*, and IS*5075*. The *mcr-1* resistance determinant was identified in IncHI2 plasmids pCFS3273-1 and pCFS3292-1, thus providing some of the earliest examples of *mcr-1* reported in Europe, and these sequences may be a representative of the early *mcr-1* plasmidome characterisation in the EU/EEA.

## 1. Introduction

*Escherichia coli* (*E. coli*) is generally considered to be commensal in the intestinal tract of mammals. However, this bacterium can be isolated from multiple points in the food chain, usually due to contamination by faecal material. The *E. coli* chromosome typically ranges in size from 4.5–5.5 Mb and displays a high degree of genomic plasticity with an open pangenome [1]. There is considerable genomic variability among commensal and pathogenic *E. coli*, and different tools for studying its population genetics have been used, including serotyping, molecular approaches for typing schemes and phylogrouping [1,2]. In cattle, *E. coli* is an aetiological agent for bovine diarrhoea but it can also be linked to 30% of mastitis infection cases [2]. These two debilitating diseases affect not only animal welfare, but also economically impact the productivity of cattle-based foods for human consumption. Even after full recovery from bovine mastitis, the milk produced by these infected animals may still contain pathogenic microorganisms, thus extending the economic loss period due to the presence of foodborne pathogens even after pasteurisation [3]. Moreover, some bacterial isolates are persistent, leading to recurrent mastitis [4]. Antimicrobial compounds are an important class of drugs for the fight against infection [5] and since their discovery early in the last century, they have had a major impact on both animal and human health alike. However, according to reports from the Centres for Disease and Prevention (CDC) about one in five resistant infections are caused by pathogens derived from food and animals [6]. Regrettably, selective pressure imposed upon various ecological niches through the misuse of antimicrobial agents has resulted in the evolution and selection of bacteria expressing resistance to one or more of these drugs [7]. However, bacteria carrying antibiotic-resistant genes are ubiquitous in nature as evidenced by commensal *E. coli* in humans with no previous exposure to antibiotics, which are reservoirs for antibiotic resistance genes [8]. Additionally, existing evidence shows that when a new compound is introduced for use, resistant bacteria can be detected soon thereafter [7]. Colistin is used as a last-resort drug to treat multidrug-resistant infections in humans, although decades of colistin use in veterinary medicine have been documented in the European Union (EU) [9]. Colistin resistance transmission was thought to be vertical due to chromosomally-mediated mechanisms; then in late 2015, horizontally acquired plasmid-mediated colistin resistance gene *mcr-1* was reported by Liu and colleagues [10], which explained the detection of this gene and its variants in different microorganisms and foodborne pathogens [11,12,13]. This stimulated the research and characterisation of plasmids carrying these virulence factors in relation to public health and genomic epidemiology. In fact, the *mcr-1* element has been described on different types of plasmids, including IncI2, IncHI2, and IncX4 that are involved in the dissemination of the resistance gene in several environmental niches and they carry additional genes for resistance to heavy metals and multiple antimicrobials [14,15]. As part of a monitoring programme on antimicrobial susceptibility, 147 *E. coli* strains were isolated from faecal samples of cattle with diarrhoea and from milk-aliquots of cattle with mastitis in both France and Germany [16]. This previous study described the presence of resistant phenotypes in the population and considering that it has been suggested that *mcr-1* has spread from food animals to humans [17,18] and the importance of comparative studies on *mcr-1* carrying isolates on a global level, in this study, the whole-genome sequences of the strains were obtained and the samples positive for the *mcr-1* gene were further investigated. The plasmid components were revealed by using the Pacific Bioscience Single Molecule Real-Time (SMRT) sequencing platform. A comparative genomic characterisation is presented and the sequenced plasmids represent one of the earliest known examples of *mcr-1* in the EU. This provides an insight into the dynamics of these horizontally acquired resistance genes.

## 2. Results and Discussion

### 2.1. Population Structure, Sequencing and Analysis of Gene Content

**Figure 1:**
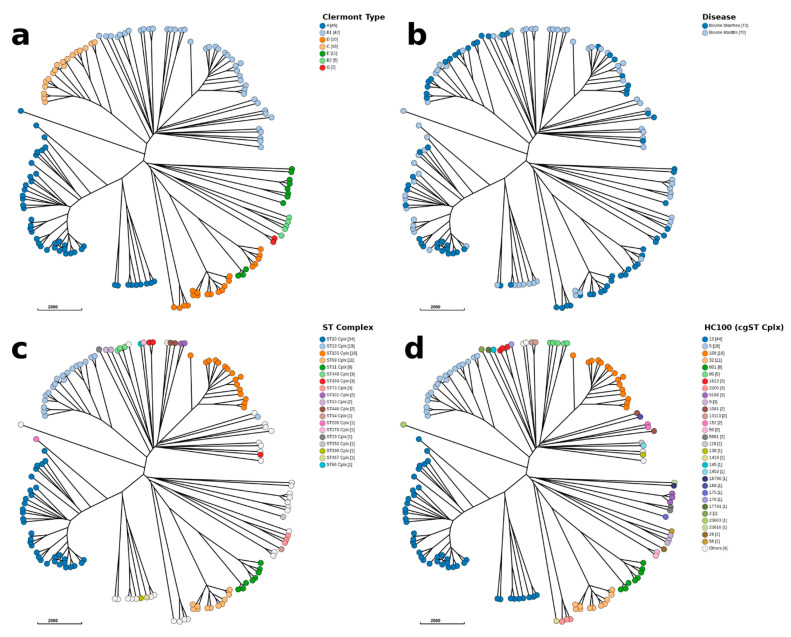
wgMLST Ninja Neighbour Joining GrapeTree. (**a**) Isolates coloured in based on the Clermont Type phylogrouping scheme. (**b**) Isolates from bovine mastitis or diarrhoea samples were broadly distributed in the phylogenetic tree. (**c**) The ST complexes were also well represented in the collection of isolates. (**d**) Separation of isolates by hierarchical clustering (HC1100) corresponds with the ST complexes as previously noted.

*E. coli* were isolated from animals presenting with diarrhoea or mastitis symptoms and subjected to whole-genome sequencing. The original collection consisted of 150 strains that were isolated from 2004 to 2010 and the raw reads from this collection were submitted to the European Nucleotide Archive (ENA) under accession number PRJEB32666. One hundred and forty-seven strains were confirmed as *E. coli* and 143 strains were included in this study, while four assemblies were discharged due to their poor quality (CFS3241, CFS3306, CFS3348, and CFS3367). The strains represented seven diverse phylogroups (A, B1, B2, C, D, E, and G) as determined by ClermontTyping in EnteroBase [19] (Figure 1a).

Bovine mastitis isolates were broadly distributed through all phylogroups, supporting evidence that there are no clearly related genotypes for a proposed bovine mastitis pathotype (Figure 1b). Sequence types ST10, ST23, and ST155 complexes were the most commonly identified (Figure 1c), reflecting the results from a study of 39 *E. coli* isolated from extraintestinal organs from livestock animals [20]. The hierarchical clustering (HierCC) based on a core genome MLST (cgMLST) with 1100 allelic distances (HC1100) mostly corresponded to ST complexes identified in the sequenced isolates, highlighting 34 different cgSTs (Figure 1d). H-antigen identification through serotype prediction showed H18 and H9 to be the most prevalent (n = 15 each) and are presented in Table S1 (Supplementary Materials).

The isolates were analysed for the presence of virulence genes through the use of the virulence factor database (VFDB) (http://www.mgc.ac.cn/VFs/, accessed on 28 March 2020), and queried by ABRicate and an *E. coli* specific database (Table S2). Three strains were positive for the presence of Shiga-toxin genes and different serotypes that have been previously reported in various food-producing animals [21,22] and humans [23]: the strain CFS3373 was predicted as serotype O88:H25 (ST58/B1/*stx1A*/*stx1vB*), the strain CFS3256 as serotype O69:H11 (ST21/B1/*stx1A*_a_/*stx1B*_1b_) and the strain CFS3265 as serotype O2:H27 (ST10/A/*stx2A*/*stx2B*). Two strains (CFS3248 and CFS3294) were predicted as serogroup O121, and O45, respectively, two of the seven (O26, O45, O103, O104, O111, O121 and O145) non-O157 serotypes that are associated with serious illness and major outbreaks [24] (Table 1).

Almost every strain in the ST69 complex, aside from *E. coli* CFS3269 and CFS3301, has *eaeX,* which encodes for an invasin/intimin-like large outer membrane protein [25]. The *eaeX* gene has also been identified in other ST69 *E. coli* [26]. According to previous reports, the increased serum survival gene *iss* and the closely related *bor* gene were commonly identified in bacteria linked with an extra-intestinal pathotype (ExPEC) and was also found in the case of bovine necrotoxigenic *E. coli* [27]. While *iss/bor* is generally associated with ExPEC, the *iss/bor* genes are some of the most commonly reported virulence genes in bovine mastitis [28] and serum resistance may play a role in bovine mammary gland colonisation [29]. The presence of *iss/bor* genes were broadly distributed in mastitis and diarrhoeic sequenced strains in this study.

Four *E. coli* isolates were either positive for *mcr-1* as informed by short reading sequencing data or were resistant to colistin by broth microdilution (data not shown), thus *E. coli* CFS3246, CFS3273, CFS3292, and CFS3313 were sequenced using the PacBio SMRT platform to provide closed genomes for a more in-depth investigation. In addition to the bacterial chromosomes, thirteen total plasmids contained in these isolates were also resolved and queried for features of interest. These included the presence of antibiotic resistance encoding genes, siderophores, and T4SS. A detailed summary of the relevant features of all thirteen plasmids is provided in Table 2.

Plasmid sizes ranged from 3.5 through to 268 kbp. Several replicon types were identified, including IncHI2 (for plasmids pCFS3273-1 and pCFS3292-1), IncF types (for plasmids pCFS3246-1, pCFS3246-2, pCFS3273-2, pCFS3292-2, and pCFS3313-1), and ColRNAI (for plasmids pCFS3246-3, pCFS3273-3, and pCFS3313-4). Various putative TA pairs were identified in these plasmids including Type II TA systems. A HigB-encoding gene flanked by hypothetical proteins was detected on plasmids pCFS3273-1 and pCFS3292-1 and these are known to stabilise extrachromosomal elements [30]. Other TA systems identified included the *phd*/*doc* TA system mapped to plasmid pCFS3292-3 and a putative TA pair within the AbrB/MazE/SpoVT family, in plasmids pCFS3246-1 and pCFS3273-2, which are regulated by conditional cooperativity [31].

To understand the distribution and variability of these plasmids, the complete plasmids were queried by PLSDB with default parameters to identify other closely related plasmids (Table S3). All of the sequenced plasmids queried revealed that the majority of related plasmids are found in *E. coli* and are globally distributed. The related IncHI2 plasmids (pCFS3273-1 and pCFS3292-1) were more widely distributed and were identified in representatives from *Salmonella enterica*, *Klebsiella* species, and *Yersiniaceae* family members.

### 2.2. Colistin Resistance Genotypes and Mobile Colistin Resistance Genes

**Figure 2:**
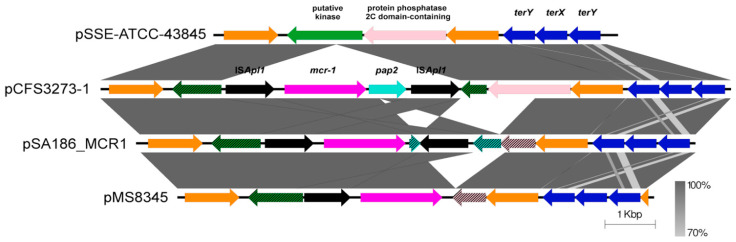
Genetic map showing the ISAp1 *mcr-1* transposon Tn*6330* in plasmid pCFS3292-1 and pCFS3273-1 compared to the reference Tn*6330* described by Li et al. in pHYEC7-*mcr1*. Each colour represents a different gene as specified in the figure. Orange colour represents “other genes”.

The *mcr-1* resistance determinant was identified in IncHI2 plasmids pCFS3273-1 and pCFS3292-1. *E. coli* CFS3273 was originally isolated in 2007 in France and *E. coli* CFS3292 was isolated in 2010 in France; thus, they provide some of the earliest examples of *mcr-1* reported in Europe. Both plasmids pCFS3273-1 (Figure 2) and pCFS3292-1 contained the tellurite resistance operon [32] located towards the distal end of the sequence.

The *mcr-1* composite transposon (IS*Apl1*-*mcr-1*-*pap2*-IS*Apl1*) in plasmids pCFS3292-1 and pCFS3273-1 was found to be identical to the previously described Tn*6330* [33]. The integration of Tn*6330* into a putative sugar kinase gene potentially silences L-arabinose utilisation [34] (Figure 2, hatched green gene). Remarkably, the synteny and sequence of the kinase and tellurite resistance loci in IncHI2 plasmids are highly conserved over several decades, being present in plasmid pSSE-ATCC-43845 from an antibiotic sensitive *Salmonella* Senftenberg originally isolated in 1941 [35]. The instability of IS*Apl1* is highlighted with the disruption of *pap2* and truncation of the protein phosphatase 2C domain-containing gene in plasmid pSA186_MCR1 [36] and loss of *pap2-*IS*Apl1* in plasmid pMS8345 [37]. Notably, some of the oldest reported *mcr-1* positive *E. coli* were cultured from the faeces of diarrhoeic veal calves in France in 2004 [38] and in 2005 [39]. Although the plasmid from the 2005 isolate was not determined by whole genome sequencing, the co-occurrence of *mcr-1* and extended spectrum β-lactamase (ESBL) resistance genes were noted within this collection. As plasmids pCFS3292-1 and pCFS3273-1 still retain both flanking copies of IS*Apl1*, these sequences represent the ancestral structure of Tn*6330*. While plasmid pCFS3273-1 does not encode ESBL, it does encode both *mcr-1* and *bla*_TEM-1_ and may be a representative of the early *mcr-1* positive plasmidome in the EU/EEA.

Both plasmids pCFS3292-1 and pCFS3273-1 were identified as IncHI2 types, which are active in acquiring antibiotic resistance-encoding genes [40], and indeed, most plasmids identified in Europe with carriage of *mcr-1* are IncHI2 types [14]. IncHI2 plasmids are recognised as important vectors for the dissemination of antimicrobial resistance markers [41] and as potentially efficient vectors for transmission of *mcr-1* [40], thereby highlighting the need to monitor the plasmidome to reduce the risk of co-selection.

When phenotypically assessed, the presence of *mcr-1* on plasmid pCFS3273-1 appeared to be sufficient in conferring resistance to colistin. In contrast, *E. coli* CFS3292 only had intermediate resistance to colistin despite the presence of *mcr-1*. Several studies reported that the expression of *mcr-1* is influenced by the nature of the different bacterial host backgrounds [40]. There is also the possibility that different genetic contexts found on these plasmids or in the bacterial isolates themselves could also account for the observed variation in colistin resistance. When overexpressed, *mcr-1* is thought to contribute to a reduction in the fitness of *E. coli*, thereby attenuating its virulent phenotype in animal models. The bacterium is challenged to find a delicate equilibrium between expression of MCR-1-mediated colistin resistance and minimising toxicity, thereby ensuring cell survival [42]. In contrast, reduced expression of *mcr-1* contributes to an increase in the susceptibility of the bacterium to colistin [43].

No horizontally acquired colistin resistance genes were identified in *E. coli* CFS3246 and CFS3313. Thus, a search for mutations in *pmrA/pmrB*, *phoP/phoQ*, and *mgrB* was conducted on all sequenced isolates. A novel A104V PmrA amino acid substitution (UniProt P30843) in the colistin susceptible *E. coli* CFS3292 was identified, and it is unknown if this mutation is linked to susceptibility to colistin in this isolate. A G53R substitution in PmrA, similar to one in *E. coli* CFS3313, was shown to confer colistin resistance in *Salmonella enterica* [44] and also identified in a number of *E. coli* isolated from Parisian patients [45]. A D149Y amino acid substitution in PmrB (UniProt P30844) was identified in *E. coli* CFS3246 [46]. In *E. coli* CFS3313, a D283G substitution [47] along with a Y358N substitution [48] was identified in PmrB. An I44L substitution in PhoP (UniProt P23836) was present in *E. coli* CFS3313 and the mutation has been previously linked to colistin resistance in *E. coli* [48]. The amino acid substitutions identified in these chromosomal genes in CFS3246 and CFS3313 are likely to contribute to the colistin resistance phenotype observed.

In the complete genomes, only *E. coli* CFS3273 encodes antimicrobial resistance genes in the chromosome. Analysis of the AMR-encoding region shows the presence of the recently described trimethoprim resistance gene, *dfrA36* [49]. The genetic context of *dfrA36* revealed that this gene was embedded in the *floR-*IS*CR2-dfrA36-sul2* element. Wüthrich et al. identified *dfrA36* with the IS*CR2* element in *E. coli* and *Acinetobacter* species in the Czech Republic, France, Germany, Israel, and the Netherlands [49]. A BLASTn analysis of the *E. coli* collection in this study found additional strains CFS3253, CFS3257, CFS3272, CFS3309, CFS3314, and CFS3325 with *dfrA36* (Table S2). Each of the 7 strains with *dfrA36* was also positive for *floR* and *sul2*, likely present on the IS*CR2* element. The *dfrA36* positive *E. coli* strains were also broadly distributed in both diarrhoea and mastitis cattle, Germany and France, and phylogroups A, B1, C, and E (Table S1).

### 2.3. Acquired Antimicrobial Resistance Genes

**Figure 3:**
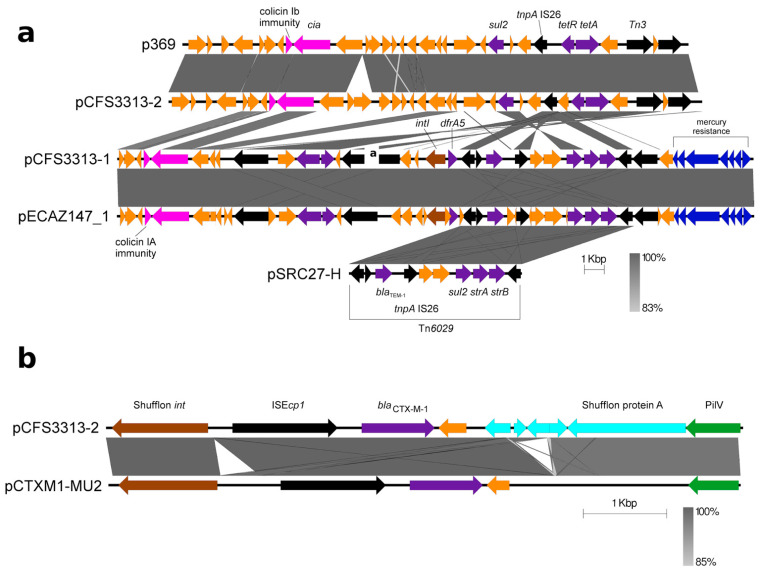
Complex AMR modules in pCFS3313-1 and pCFS3313-2. (**a**) Highlighted in the figure are three selected BLAST hits—plasmids p369, pECAZ147_1, and pSRC27-H. Plasmid p369 showed a higher degree of similarity with plasmid pCFS3313-2 but comparatively less similarity with plasmid pCFS3313-1, which matched closely with plasmid pECAZ147_1. An intact Tn*6029* module is present in both plasmids pCFS3313-1 and pECAZ147_1. Colour scheme: antimicrobial resistance genes (purple), heavy metal resistance genes (blue), transposons and insertion elements (black), colicin resistance (pink), and integrases (brown). (**b**) The IncI1-blaCTXM-1 plasmid pCTXM1-MU2 shows high synteny with plasmid pCFS3313-2. The ISEcp1 module is present in both plasmids. Colour scheme: antimicrobial resistance genes (purple), shufflon genes (light blue), transposons and insertion elements (black), and integrases (brown). Other genes are represented in orange.

**Figure 4:**
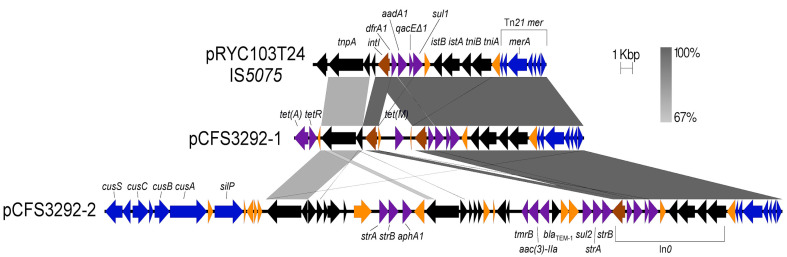
Complex AMR-encoding region in plasmids pCFS3292-1, pCFS3292-2, and IS*5075* from plasmid pRYC103T24. Tetracycline resistance *tet*(B) and *tetR* are associated with the IS*5075* in plasmid pCFS3292-1 as well as the uncommon *tet*(M) resistance gene. Plasmid pCFS3292-2 contains an additional complex AMR module with *bla*_TEM-1_ and two identical copies of *strAB*. Colour scheme: antimicrobial resistance genes (purple), heavy metal resistance genes (blue), transposons and insertion elements (black), and integrases (brown). Other genes are represented in orange.

Other antimicrobial resistance-encoding genes were identified among the sequenced plasmids in all four closed *E. coli* genomes. Different β-lactamase-encoding genes were identified in several plasmids with plasmids pCFS3273-1, pCFS3292-2, and pCFS3313-1 encoding *bla*_TEM-1_, while plasmid pCFS3273-2 encoded a *bla*_TEM-1_ variant. The ESBL-encoding *bla*_CTX-M-1_ gene was identified on plasmid pCFS3313-2 (Table 2). In addition, these plasmids also included *dfrA* encoding resistance to trimethoprim, *tet* encoding tetracycline resistance and sulphonamide resistance genes (Figure 3a,b and Figure 4).

The AMR-containing loci from the sequenced plasmids were used in BLASTn searches to identify similar regions. Plasmid p369, which was originally isolated from 7-day old broiler chicks [50], and plasmid pCFS3313-2 appeared to show extensive sequence conservation over regions greater than 100-kbp (Accession KT779550.1, Table S3). Both were typed as IncI1 plasmids containing *bla*_CTX-M-1_, *sul2* encoding sulphonamide resistance*,* and *tet*(A) resistance-encoding genes. Similarly, for plasmids pCFS3313-1 and pECAZ147_1 (cultured from an *E. coli* isolated from human faecal samples), these plasmids showed extensive conservation over a region of some 150 kbp. More limited sequence similarities were identified between plasmids pCFS3313-2 and pCFS3313-1, and again between pECAZ147_1 (Figure 3a). Plasmid pCFS3313-2 shows high similarity with a persistent and broadly distributed ESBL-encoding plasmid pCTXM1-MU2 [51] (Accession MF152729.1, Table S3). The IS*Ecp1* element that encodes *bla*_CTX-M-1_ was found in both plasmids and IncI1 plasmids carrying these elements were reported to be common in Europe (Figure 3b).

Plasmids p369 and pCFS3313-2 contained a *cia*-encoding gene (Colicin Ia gene), a channel-forming transmembrane bacteriocin. These gene products are cytotoxic to other bacteria and potentially confer fitness by eliminating competition. A module located distally to the latter marker in plasmid pCFS3313-2, contained *tnpA-tetR-tet*(A)*-pecM* and was present on plasmids p369 and pECAZ147_1 (Figure 3a). This module was also identified in plasmid pCFS3313-1, but it was integrated at a different locus. Plasmid pCFS3313-1 additionally contained a Tn*21* derivative with an atypical integron (encoding *drfA5*) [52], a complex multidrug resistance transposon Tn*6029* (encoding *bla*_TEM-1_, *strAB*, and *sul2*) and a mercury resistance module (*merRTPCAD*) that was nearly identical to one found in plasmid pECAZ147_1. This module with the atypical integron and Tn*6029*-like resistance transposon derivative was found to be similar to one initially identified in plasmid pO26-CRL_125_; however, it does not encode a*phA1*.

Plasmid pCFS3292-1 contained a single copy of *tet*(M), transposon Tn*21* encoding mercury resistance, and *dfrA1-aadA1-qacEΔ1-sul1* within an In*0* module, which encodes resistance to trimethoprim, aminoglycosides, quaternary ammonium compounds, and sulphonamides, respectively (Figure 4). The presence of *tet*(M) is unusual in that it is rarely associated with Gram-negative bacteria; however, it has been reported in clinical isolates of *E. coli* [53]. The In*0* and Tn*21 mer* modules comprise IS*5075* and when sequence similarities between plasmids pRYC103T24, pCFS3292-1, and pCFS3292-2 were considered, conserved synteny and nucleotide sequences were noted. When compared to IS*5075*, additional integration of AMR-encoding genes in pCFS3292-1 and pCFS3292-2 between the *intI* integron and *tnpA* were observed (Figure 4). Specifically, in plasmid pCFS3292-2, a complex module encoding two copies of *strA, strB,* and *aphA1* along with single copies of *tmrB, aac(3)-IIa, bla*_TEM-1_*,* and *sul2* was present in this region. Plasmid pCFS3292-2 also contained the copper cation efflux *cus*-encoding cluster (*silP-cusA-cusB-cusF-cusC-cusR-silE*), which is similar to a recently described copper homeostasis and silver resistance island (CHASRI); however, it lacks the *pco* plasmid-borne copper resistance system [54].

**Figure 5:**
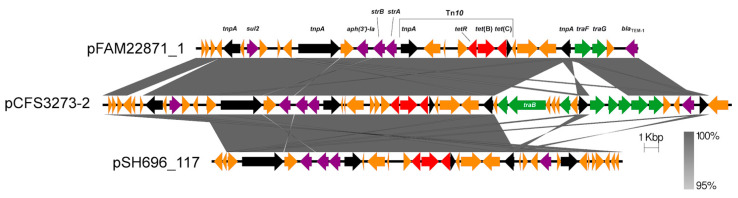
Complex AMR region between plasmids pFAM22871_1, pCFS3273-2, and pSH696_117. High conservation of a complex antimicrobial resistance gene region with the presence of Tn*10* in plasmids pCFS3272-2. Extensive deletion of the *tra* region is observed in plasmid pFAM22871_1. Colour scheme: antimicrobial resistance genes (purple), tetracycline resistance genes (red), transposons and insertion elements (black), and genes of interest (green). Other genes are represented in orange.

The comparative map of plasmid pCFS3273-2 with plasmids pFAM22871_1 isolated from raw milk cheese [55] and plasmid pSH696_117 (*S. enterica* serovar Heidelberg isolated from turkey carcass swabs taken at a Midwestern American processing facility in 2000 [56] show high conservation of a complex antibiotic resistance region (Figure 5).

Additionally, plasmids pCFS3273-2 and pFAM22871_1 encode *bla*_TEM-1_, located towards the distal end. Other antimicrobial resistance-encoding genes identified included *aph(3′)-Ia, strAB,* and *sul2.* The Tn*10* module encoding tetracycline resistance genes *tet*(B)*, tet*(C) and *tetR* was also present in all three plasmids. Some conserved genes including *sul2*, encoding resistance to sulphonamides, were mapped on plasmids pFAM22871_1 and pCFS3273-2. Plasmid pCFS3273-2 also contained a putative VirB4 type IV ATPase/TraB-encoding gene, which was not present in any of the other two comparative isolates. However, both plasmids, pCFS3273-2 and pFAM22871_1 harboured the IncF plasmid conjugative transfer genes *traF* and *traG*. The instability of the IS*26tnpA* in the *tra-*encoding genomic region may have resulted in the deletion of this operon in plasmid pFAM22871_1 as the *tnpA* interrupts a *traO* gene (not shown). The presence of heavy metal resistance genes in the sequenced plasmids was noted (Figure 2, Figure 3 and Figure 4). A recent study on zinc contamination within deposited sediment in a pond showed there was a positive correlation of heavy metal contamination with resistance to clinically relevant antibiotics [57]. Increased detection of the class 1 integron integrase gene was observed in correlation with zinc. Heavy metal contamination may play a role in the co-selection of plasmids that may harbour antibiotic resistance genes [58], especially since contaminated environments that pre-date the antibiotic era were enriched for antibiotic resistant bacteria. As previously noted, the tellurite resistance operon is remarkably stable in IncHI2 plasmids over a number of decades [35].

### 2.4. Genetic Characterisation of Plasmids pCFS3313-1 and pCFS3273-2 Containing Siderophore-Encoding Genes and ColV Genes

**Figure 6:**
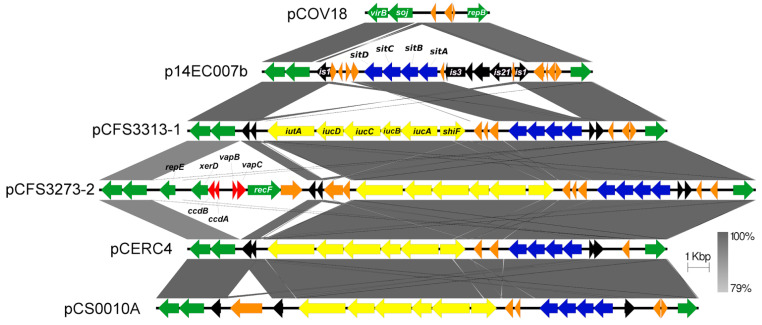
Siderophore units identified in plasmids pCFS3313-1 and pCFS3273-2. Highlighted in the figure are four selected BLAST hits—plasmids pCOV18, p14EC007b, pCERC4, and pCS0010A. The first two plasmids show a high level of similarity in the flanking regions while the latter two show similarity across the siderophore containing units along with the flanking regions. Colour scheme: siderophore (yellow), metal transporters (blue), transposons and insertion elements (black), toxins/antitoxins (red), and genes of interest (green). Other genes are represented in orange.

Plasmids pCFS3313-1 and pCFS3273-2 encode the *iutADCB*-*iucA*-*shiF* aerobactin siderophore and manganese/iron transport *sitABCD* operons (Figure 6).

The *sitABCD* operon in both plasmids is identical with a plasmid from an avian pathogenic *E. coli* [59]. Plasmid pCFS3313-1 is closely related to the virulence plasmid pAPEC-1 (NC_011980.1, Table S3). BLAST searches identified a number of plasmids that also contained siderophore-encoding regions (as in the case of plasmids pCERC4 and pCS0010A, identified previously in Australian adults and *Salmonella* serovars cultured from broiler chickens, respectively) along with other loci (including plasmids p14EC007b and pCOV18, which were identified in French broilers) [60]. The linear map of these plasmids highlights the conserved siderophore region amongst all sequences except for plasmid pCOV18, which lacks the siderophore module but shows conservation of the flanking virulence gene-encoding regions (Figure 6). Siderophores function as metal-chelating agents and are of interest due to being expressed under limiting iron conditions. They primarily scavenge iron and form complexes with essential metal ions to increase the bioavailability to its host. The *sitABCD* genes have also been linked to resistance to oxidative stress-mediated by metal cofactors [61]. The membrane receptors expressed by bacteria recognise the complex, which is then transported across the periplasmic space through the ATP-binding cassette (ABC) transport system to reach the cytoplasm where iron is released via a reduction mechanism. In terms of the aerobactin unit and the *sit* operon, both showed a high level of similarity to plasmids pCERC4 and pCS0010A wherein the IS*6110* seems to be conserved throughout the sequences, preceding the siderophore-encoding genes (*iutA, iucD, iucC, iucA,* and the *shiF* membrane transport protein) (Figure 6). In addition to the siderophore modules, these plasmids also shared the genes involved in maintenance and stability regions. However, the flanking regions appear to be largely conserved between the two plasmids. Besides the sporulation initiation inhibitor protein Soj present and conserved between the plasmid pCOV18 and others, there was also conservation in the CcdB-CcdA toxin-antitoxin system shared between plasmids pCOV18 and pCFS3273-2.

The backbone of these plasmids contains IncFII-2 and IncFIB-1 replicons and the conserved ColV virulence region; however, plasmid pCERC4 also contains an additional ColIa-encoding determinant [60]. Plasmids pCFS3313-1 and pCFS3273-2 are typical of ColV plasmids in that they contain the ColV virulence genes, they are large and heterogeneous in size and genetic composition, contain IncFI replicons, and possess conjugative modules [62,63]. ColV plasmids have been recognised as a defining trait of avian pathogenic *E. coli* (APEC) in colonising and infecting commercial broiler chickens and turkeys. They are generally thought to have arisen from conjugative plasmids that acquired colicin V and the virulence genes by recombination, and the presence of these genes in one genetic structure confers a selective advantage to *E. coli*. ColV plasmids in *E. coli* ST131-H22 have been found in human clinical *E. coli,* which provides evidence of historical zoonotic transmission from poultry to humans [64]. While they are predominantly *traT* negative, they may be mobilised *in trans* by other conjugable plasmids among different *E. coli* strains, thus endowing these strains simultaneously with several virulence-related properties.

### 2.5. Genetic Characterisation and Analysis of Plasmid pCFS3313-3 Containing A Type IV Secretion System (T4SS)

**Figure 7:**
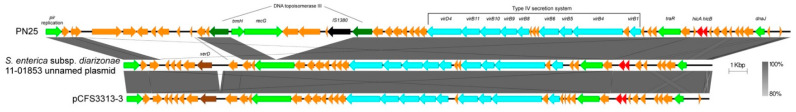
Linear plasmid map showing the T4SS units in plasmid pCFS3313-3. Conservation and synteny of IncX4 plasmid pCFS3313-3 with plasmid PN25 and an unnamed plasmid from *S. enterica* subsp. *diarizonae* 11-01583. Along with the similarity in T4SS, the plasmids also shared similar replicase and transposon elements denoting a higher degree of conservation. Colour scheme: transposons and insertion elements (black), integrons and integrases (brown), toxins/antitoxins (red), virulence genes (T4SS unit) (cyan), and genes of interest (green).

Of the three plasmids contained in *E. coli* CFS3313, plasmid pCFS3313-3 was of interest as it contained a full T4SS. The latter are protein complexes that are located within the bacterial cell envelope. Gram-negative bacteria use these systems to translocate a variety of virulence factors into the host cell. T4SSs are also known to mediate horizontal gene transfer, which in turn, facilitates the adaptation of the bacterium to a specific environmental niche, and which can also contribute to the dissemination of antibiotic resistance-encoding genes among bacteria [65]. BLAST comparative analysis of plasmid pCFS3313-3 identified IncX4 type plasmid PN25 [42] along with an unnamed plasmid from *Salmonella enterica* subsp. *diarizonae* strain 11-01853 isolated from a fatal septicaemia case [66] (Figure 7).

Notably, plasmid PN25 contains the *mcr-1* gene, a feature not shared with plasmid pCFS3313-3. Along with the similarity in T4SS, the plasmids also shared similar replicase and transposon elements, which depicts a higher degree of conservation in the T4SS flanking regions amongst the plasmids carrying these genes. A Mash screen of plasmid pCFS3313-3 in the PLDSB revealed other closely related IncX4 plasmids [67] (Table S3). The stability and high conservation of IncX4 plasmid backbones were noted in variants that carry *bla*_CTX-M-14b_ and *mcr-1* [68,69].

## 3. Conclusions

Pathotypes of *E. coli* can be linked epidemiologically to the modern food chain and some types of foodborne *E. coli* have links with human extra-intestinal infections, a feature that underscores the public health relevance arising from the use of antimicrobial compounds in food animal production [64]. Not only can these isolates cause infections in humans, they are also pathogenic for various animals, including those harvested for food [70]. Similarly, this bacterial genus is also an aetiological agent in cases of mastitis in cattle. When necessary, antimicrobial compounds are used for chemotherapy as well as for production purposes. Concerns have been expressed about the selective pressure that this practice may impose on various ecological niches, such as the microbiome of food-producing animals. This in turn may lead to the emergence of isolates that become resistant to those very compounds that are used to treat infections. Understanding the nature of the bacterial plasmidome provides another risk assessment-based approach to protecting public health and improving food safety protocols.

## 4. Materials and Methods

### 4.1. Bacterial Cultures, Genomics DNA Purification and Sample Preparation for Sequencing

Bacteria were isolated from samples taken from animals suffering from digestive (diarrhoea) and mastitis pathologies as part of an ongoing inhouse monitoring program on antimicrobial resistance. All the samples were maintained on Cary-Blair transport medium (Oxoid, Basingstoke, UK) and then stored in a cool box. Only one sample was taken from each animal and the corresponding bacterial strain was included in the study. The isolates were identified to species level using the API biochemical identification system (bioMérieux, France). Isolates were stored at −80 °C in lysogeny broth (LB) (Sigma Aldrich, Arklow, Ireland) supplemented with 15% (*v/v*) glycerol. Incubation at 37 °C for 24 h in Mueller-Hinton (MH) agar (Sigma-Aldrich, Arklow, Ireland) was used to confirm the monoculture and single colonies were inoculated in 5 mL of MH broth (Sigma-Aldrich, Arklow, Ireland) and cultured overnight for 18 h at 37 °C with orbital shaking at 200 RPM.

Total genomic DNA (gDNA) was purified from overnight cultures of bacterial isolates using the DNeasy UltraClean Microbial Kit (Qiagen, Manchester, UK). Quantification and quality assessment of gDNA was assessed using Qubit (Thermo Scientific, Dublin, Ireland) and NanoDrop (Thermo Scientific, Dublin, Ireland). For each sample, 2 ng/µL of gDNA was used as a template for library preparation, using the Nextera XT Library Preparation kit (V3 300-cycles) and loaded onto the Illumina MiSeq platform. 

For SMRT sequencing on the PacBio RS II platform, libraries using 6 μg gDNA were sheared to a size of 20 kb using g-TUBEs (Covaris Inc., Brighton, UK) according to the manufacturer’s instructions. The template libraries were constructed using SMRTbell Template Prep Kit 1.0 with the 20 kb insert library protocol (Pacific Biosciences, Menlo Park, USA) and sequenced using the P6-C4 chemistry on 1 or 2 single-molecule real-time (SMRT) cell with a 360-minute collection protocol along with Stage Start.

### 4.2. Bioinformatics Analysis

Raw read quality was assessed with FastQC (version 0.11.7) (http://www.bioinformatics.babraham.ac.uk/projects/fastqc/, accessed on 28 March 2020) and low-quality sequences were trimmed using Trimmomatic (version 0.39) [71]. Trimmed, paired reads were *de-novo* assembled using SPAdes (version 3.13.0) [72] and the resulting contigs were quality-assessed with QUAST (version 5.0.2) [73]. Analysis of the PacBio data was implemented using SMRT Analysis 2.3.0. The best de novo assembly was empirically determined and manually finished by the Hierarchical Genome Assembly Process (HGAP 3.0) and Canu [74]. Genomes were checked manually for even sequencing coverage. Afterwards the interim consensus sequence was used to determine the final consensus and accuracy scores using the Quiver consensus algorithm (16). Genomes were uploaded to NCBI under accessions CP026929-CP026942 and CP053652-CP053654 and then annotated by the NCBI Prokaryotic Genome Annotation Pipeline (PGAP) [75]. EnteroBase [76] was used to curate strains from the whole-genome sequencing project (BioProject Accession PRJEB32666). A Ninja neighbour-joining (Ninja NJ) algorithm [77] was used to reconstruct a GrapeTree phylogeny tree [78] utilising the whole genome multi-locus sequence typing (wgMLST) scheme in EnteroBase. Raw reads were automatically imported into EnteroBase and assembled through the default pipeline. Serotype prediction and sequence typing (Achtman 7 gene MLST) were performed within EnteroBase obtaining for each isolate, the allelic profile for generating the sequence type (ST). Core genome sequence types (cgSTs) were obtained through hierarchical clustering (HierCC) following the standard parameters on EnteroBase.

Additional metadata and sample IDs are available in Table S1. Abricate v1.0.0 (https://github.com/tseemann/abricate, accessed on 28 March 2020) was used to query genome assemblies with the ResFinder database and VFDB [79,80]. The MEGARes [81], PlasmidFinder [82], and the *E. coli* specific virulence databases were also queried by Abricate. The combined results into a gene presence/absence matrix and the correspondent values indicating nucleotide percent identity are presented in Table S2. The plasmid database, PLSDB v. 2020_03_04, was used in a sequence search for related plasmids within the PacBio sequenced genome datasets [83] (Table S3). Mash distance (v. 2.1.1 on PLSDB, default parameters at maximum *p*-value = 0.1 and maximum distance = 0.1) was used to identify closely related plasmids [84] and EasyFig v2.2.2 was used to generate comparative alignments of sequences [85].

### 4.3. Antimicrobial Susceptibility Test (AST)

Antimicrobial Susceptibility testing was carried out on all 143 isolates in accordance with Clinical and Laboratory Standards Institute guidelines for disk diffusion [86]. A total of 10 antibiotics were used covering 8 antimicrobial classes and included: amoxicillin (10 µg), amoxicillin/clavulanic acid (20/10 µg), gentamicin (10 µg), cephalothin (30 µg), ciprofloxacin (5 µg), florfenicol (30 µg), marbofloxacin (5 µg), nalidixic acid (30 µg), tetracycline (30 µg), trimethoprim-sulfamethoxazole (1.25/3.75 µg). Each antimicrobial compound was tested in triplicate and the average zone size was used to determine resistance/sensitivity. *E. coli* ATCC™25922 was used as a control strain.

## Figures and Tables

**Table 1:** Strains’ non-O157 serotypes associated with serious illness and major outbreaks.

Strain Id	Clermont Type	Serotype Prediction	Shiga-Toxin Genes
CFS3373	B1	O88:H25	*stx1A*/*stx1vB*
CFS3248	E	O121:H15	n.d.
CFS3294	B1	O45:H16	n.d.

**Table 2:** Relevant features of all thirteen plasmids. The nucleotide sequence lengths are provided along with the Inc- and pMLST-types for plasmids and the associated toxin-antitoxin (TA) systems encoded by the plasmids.

Isolate	Plasmid	Accession	Size (bp)	Plasmid Type	β β-Lactamase(s)	Other Resistance Genes	Toxin/ Antitoxin Family
CFS3246	pCFS3246-1	CP026930	129,230	IncFIB/IncFII	TEM-1B	*aph(3′′)-Ib, aph(6)-Id*	VapB/sta1
	pCFS3246-2	CP026931	62,471	IncFII	n.d. ^1^	n.d.	HokB/YafN
	pCFS3246-3	CP053652	5279	ColRNAI_1	n.d.	n.d.	n.d.
CFS3273	pCFS3273-1	CP026933	268,665	IncHI2/ IncHI2A/IncQ1	TEM-1A	*aac(3)-IIa, aadA1* (2X)*, aadA2b, aph(3′)-Ia, aph(3′′)-Ib* (2X)*, aph(6)-Id* (2X)*, catA1, cmlA1, dfrA1, mcr-1.1, sul1, sul2, sul3, tet*(A)	HipB/HigB-1/HokB
	pCFS3273-2	CP026934	145,001	IncFIA/IncFIB/ IncFII	TEM-1B	*aph(3′′)-Ib, aph(3′)-Ia, aph(6)-Id, sul2, tet*(B)	Type II CcdA/CcdB/VapB/RelE2
	pCFS3273-3	CP053653	6648	ColRNAI_1	n.d.	n.d.	n.d.
CFS3292	pCFS3292-1	CP026936	192,449	IncHI2/IncHI2A		*aadA1, dfrA1, mcr-1.1, sul1*, *tet*(A), *tet*(M)	Type II (HigB/HigA)
	pCFS3292-2	CP026937	131,954	IncFIB/IncQ1	TEM-1A	*aac(3)-IIa, aadA1, aph(3′′)-Ib* (2X), *aph(3′)-Ia, aph(6)-Id* (2X), *dfrA1, sul1, sul2*	yafN/PemI
	pCFS3292-3	CP026938	99,789	p0111_1	n.d.	n.d.	doc
CFS3313	pCFS3313-1	CP026940	155,171	IncFIB/IncFII /IncQ1	TEM-1B	*aph(3′′)-Ib, aph(6)-Id, dfrA5, sul2, tet*(A)	relE2/apxIB/tdeA
	pCFS3313-2	CP026941	111,822	IncI1-I	CTX-M-1	*sul2, tet*(A)	relE2/tcpT/tcpE
	pCFS3313-3	CP026942	31,764	IncX4_1	n.d.	n.d.	n.d.
	pCFS3313-4	CP053654	3499	ColRNAI_1?	n.d.	n.d.	n.d.

^1^ n.d. = not detected.

## Data Availability

The raw data and the nucleotide sequences are accessible as a BioProject in the database https://www.ncbi.nlm.nih.gov/bioproject/PRJEB32666/ (accessed on 28 March 2020). Accession number PRJEB32666, ID: 544224; containing BioSamples presented in this study and SRA.

## Supplementary Materials

The following are available online at https://www.mdpi.com/article/10.3390/antibiotics10091041/s1, Table S1: Additional metadata, ID and results of typing schemes, Table S2: Summary results of genes detected queried by ABRicate, Table S3: Closed related plasmid identified and queried by PLSDB with default parameters.
